# Comparing risk assessment methods for work-related musculoskeletal disorders with in vivo joint loads during manual materials handling

**DOI:** 10.1038/s41598-024-56580-7

**Published:** 2024-03-13

**Authors:** Christopher Brandl, Alwina Bender, Tim Schmachtenberg, Jörn Dymke, Philipp Damm

**Affiliations:** 1 Institute of Industrial Engineering and Ergonomics, RWTH Aachen University, Eilfschornsteinstr. 27, 52062 Aachen, Germany; 2 Fraunhofer Institute for Communication, Information Processing and Ergonomics FKIE, Aachen, Germany; 3 Julius Wolff Institute of Biomechanics and Musculoskeletal Regeneration, Berlin Institute of Health at Charité – Universitätsmedizin Berlin, Berlin, Germany

**Keywords:** Ergonomics, Musculoskeletal diseases, Posture, Vertebral body, Hip joint, Knee joint, Risk factors, Occupational health, Mechanical engineering, Bone quality and biomechanics

## Abstract

The validity of observational methods in ergonomics is still challenging research. Criterion validity in terms of concurrent validity is the most commonly studied. However, studies comparing observational methods with biomechanical values are rare. Thus, the aim of this study is to compare the Ovako Working Posture Analysing System (OWAS) and the Rapid Entire Body Assessment (REBA) with in vivo load measurements at hip, spine, and knee during stoop and squat lifting of 14 participants. The results reveal that OWAS and REBA action levels (AL) can distinguish between different in vivo load measurements during manual lifting. However, the results also reveal that the same OWAS- and REBA-AL do not necessarily provide equal mean values of in vivo load measurements. For example, resultant contact force in the vertebral body replacement for squat lifting ranged from 57% body weight (%BW) in OWAS-AL1 to 138%BW in OWAS-AL3 compared to 46%BW in REBA-AL0 and 173%BW in REBA-AL3. Furthermore, the results suggest that the performed squat lifting techniques had a higher risk for work-related musculoskeletal disorders than the performed stoop lifting techniques.

## Introduction

The high prevalence of work-related musculoskeletal disorders (WMSD) is challenging companies and employees equally. Companies notice negative effects, for example, through incapacity to work, loss of productivity and delay in deadlines. Employees who develop from musculoskeletal disorders, for example, are restricted in their body movements, suffer from pain, require long-term therapy and are at high risk of no longer being able to perform their learned profession due to injury. Ergonomic work design and occupational interventions are commonly used for prevention of WMSD^1–3^. Thereby, ergonomic methods provide a framework for assessing exposure to related risk factors. The wide range of methods available for various requirements of operational practice are still subject to many limitations, especially concerning validity^4–8^. The Ovako Working Posture Analysing System (OWAS) as defined by Karhu et al.^9^ and the Rapid Entire Body Assessment (REBA) as defined by Hignett and McAtamney^10^ are frequently used^11^ and therefore focused in this paper. OWAS was originally developed in steel industry by applying work sampling approaches. REBA's basic idea was adapted from the Rapid Upper Limb Assessment^12^. Both methods were designed for a quick and easy evaluation of postural loads, so far used in several occupations^11,13^, and today known to be general methods for an ergonomic assessment during different working conditions.

Compared to OWAS, REBA is designed to be a more sensitive approach from a conceptual point of view because it has more categories of observations and results. A latest comparison of OWAS and REBA in terms of validity and other criteria is available^11^. However, the proof of validity was and remains a challenging task, as Li and Buckle^6^ already stated aptly. The concept of validity includes several aspects, however, criterion validity in terms of concurrent validity is the most commonly studied^8^. More precisely, the agreement of an ergonomic method with a method considered to be more valid is studied. However, many other studies compare observational methods among themselves rather than validate them and, therefore, cannot be used to draw conclusions regarding criterion validity. Studies investigating concurrent validity were performed by comparing the results of OWAS and REBA with discomfort ratings^14–18^, maximum holding times^19^, biomechanical models^15,20^, electrical muscle activity^17,21^, and expert evaluations^22,23^.

This study aims to increase knowledge on the validity of observational methods by comparing OWAS and REBA with in vivo load measurements at hip, spine, and knee.

## Methods and materials

However, in vivo load measurements are complex and limited to a small number of people worldwide. Therefore, the validation was performed based on data that have already been published partly and examples made available on www.orthoload.com as well as on data that have not yet been published. Since in vivo load measurements are limited to one joint per participant, the database was searched retrospective for common work activities performed from all participants equally. A manual materials handling (MMH) activity was found to validate OWAS and REBA with in vivo load measurements. Since OWAS and REBA are known as general methods, they should also be able to evaluate manual lifting tasks, although there are especially designed methods available for the specific application, such as the well-known National Institute for Occupational Safety and Health of the United States of America (NIOSH) lifting equation^24^. However, the NIOSH lifting equation is not intended to evaluate differences between lifting techniques^25^, whereas OWAS and REBA generally enable such an evaluation. Observational methods assess WMSD risk largely based on biomechanical exposures, such as body angles, load conditions, and temporal characteristics. In vivo load measurements capture forces and moments directly in human joints and thus represent the direct joint load. Thus, in vivo load measurements can be considered as a valid basis for criterion validation of the observational methods. The purpose of this study is to investigate whether OWAS and REBA can distinguish between different in vivo load measurements of hip, spine, and knee in similar levels of condition-related risk assessment, such as would result using the NIOSH lifting equation when evaluation different lifting techniques for the same MMH activity.

The implantation and the study protocols were approved by the institutional review board of the Charité – Universitätsmedizin Berlin (EA2/057/09; EA/069/06; EA2/057/09) and registered at the ‘German Clinical Trials Register’ (DRKS00000563, DRKS00000606). All patients gave written informed consent prior to participation in these studies, in which they agreed to implantation of the instrumented implants, in vivo load measurements and the publication of their images. All methods were performed in accordance with the relevant guidelines and regulations. The funders played no role in the design, conduct, or reporting of this study.

### Participants

All participants in this study belong to a unique and worldwide small group of patients who received different telemetric implants during joint replacement. Built-in telemetry enables these implants to measure forces and moments within the artificial joint or the vertebral body replacement during various activities. Telemetric implants have been developed and tested over several years; details are published and summarized in Sect. Telemetric implants. A total of 14 participants (11 male, 3 female) participated in the study. Each participant was provided with exactly one joint implant for in vivo load measurements. In this study in vivo load measurements of eight telemetric hip implants (HI), four telemetric vertebral body replacement (VBR), and two telemetric knee implants (KI) are analyzed. Indication of all HI participants was coxarthrosis. Participants with VBR had a A3-type vertebral compression fracture of the L1 vertebral body (n = 3) and of the L3 vertebral body (n = 1) as defined by Magerl et al.^26^. Indication of the participants with KI was gonarthrosis. Further details of the participants are summarized in Table 1.

**Table 1:** Participant information.

Implant	Implant placement	Participant	Gender	Age	Weight (kg)	Height (m)	Age at implantation
Hip implant	Right	H2R	Male	62	77	1.72	61
	Left	H3L	Male	62	91	1.68	59
	Left	H4L	Male	55	82	1.78	50
	Left	H5L	Female	64	88	1.68	62
	Right	H7R	Male	53	93	1.79	52
	Left	H8L	Male	56	89	1.78	55
	Left	H9L	Male	56	124	1.81	54
	Right	H10R	Female	54	99	1.62	53
Vertebral body replacement	Vertebra L3	WP1	Male	66	67	N/A	62
	Vertebra L3	WP2	Male	72	73	N/A	71
	Vertebra L1	WP4	Male	67	59	1.70	64
	Vertebra L3	WP5	Male	69	64	1.80	67
Knee implant	Right	K4R	Female	69	108	1.70	63
	Left	K9L	Male	80	109	1.66	75

### Telemetric implants

Detailed descriptions of the HI^27^, VBR^28^, KI^29^, and the external measurement system^30^ have previously been published. HI (CTW, Merete Medical, Berlin, Germany), VBR (SYNEX, Synthes Inc., Bettlach, Switzerland), and KI (INNEX FIXUC, Zimmer GmbH, Winterthur, Switzerland) were equipped with a telemetry unit, semiconductor strain gauges (KSP 1–350-E4, Kyowa, Japan) and an inductive power supply. Telemetry circuit, strain gauges and induction coil are fixed in the hollow implant, who is sealed hermetically. During the measurement the participants are wearing an external induction coil around the joint (HI and KI) or around the upper body (VBR). The strain gauge signals are transferred wireless to the external receiver at radio frequency via an antenna inside of the implant. Via a 6 × 6 calibration matrix, six semiconductor strain gauges deliver three force and three moment directions acting on the implant with an accuracy of about 2%^27–29,31^. Telemetric load signals and video of the participant are recorded simultaneously.

### Experimental design and procedure

As mentioned before, in this study in vivo load measurements are limited to one joint per person. The internal database was searched retrospective for a suitable work activity that all participants performed equally and at least one year postoperatively. The in vivo load data from eight patients with HI and four patients with VBR in this study focusing on resultant forces in the implant and upper body inclination was previously published^32^, whereas the in vivo load data of the two knee patients were not published before The procedure for in vivo load measurement were already published and extensively described^27–29,31^. For the validation, a manual materials lifting task was selected in which a box weighing 10 kg was lifted frontal from the floor to the waist and then placed down again. Each participant had to perform a lifting technique with straight knees (stoop lifting) and with bent knees (squat lifting). To avoid any constraints, the participants were generally given no further instructions about how to perform the exercise (e.g., choosing their self-selected lifting speed). The same lifting technique was performed 4 to 8 times by each participant. For each frame, in vivo load measurements were compared with action levels of OWAS and REBA retrospective classified based on the synchronous videos.

OWAS and REBA are observational methods for assessing exposure to risk factors for work-related musculoskeletal disorders based on work sampling for several observations. As a result of the methods users get so-called action levels (AL), which represent the risk of posture for work-related musculoskeletal disorders and designate the urgency of actions. The original OWAS describes a full body posture by classifying four back postures, three arm postures, seven leg postures and three categories of load or use of force. These postures are combined to a four-digit code, which is assigned to one of the four action levels (AL). The OWAS-AL ranges from AL1 (posture is acceptable and therefore working postures do not need to be corrected) to AL4 (posture has a very harmful effect on the musculoskeletal system and therefore corrective measures should be taken immediately). REBA scores the postures of trunk, neck, legs, upper arms, lower arms and wrists and information about load or force, coupling and activity. The REBA score is then calculated based on a simple mathematical formula and assigned to one of five REBA-AL. The REBA-AL ranges from AL0 (posture risk is negligible and no action is necessary) to AL4 (posture risk is very high and action is necessary immediately). Good descriptions of the application of OWAS and REBA can be found in Brandl et al.^33^ and Hignett and McAtamney^10^.

### Data recording and processing

Video and telemetry data in this study were recorded synchronously and stored on videotapes using a MiniDV tape recorder Sony GV-D1000 (Japan) and camcorder Panasonic NV-GS400 (Japan). The sampling rate was 50 frames per second. The telemetric signal was stored on the audio track of the videotape. The resultant in vivo joint contact forces (F_res_) acting on the implant and the in vivo joint torsional torques (M_tors_) around the implant reflecting the main load situation at the stem-bone interface are analyzed. The forces and the moments measured with a telemetry system in the implant coordinate system are transformed into the bone related coordinate system (x_b_, y_b_, z_b_) using angles measured in computer tomography^34^. F_res_ is calculated from three forces in the bone coordinate system F_x_, F_y_ and F_z_ by the following equation.

$${F}_{res}= \sqrt{{F}_{x}^{2}+ {F}_{y}^{2}+ {F}_{z}^{2}}$$

**Figure 1:**
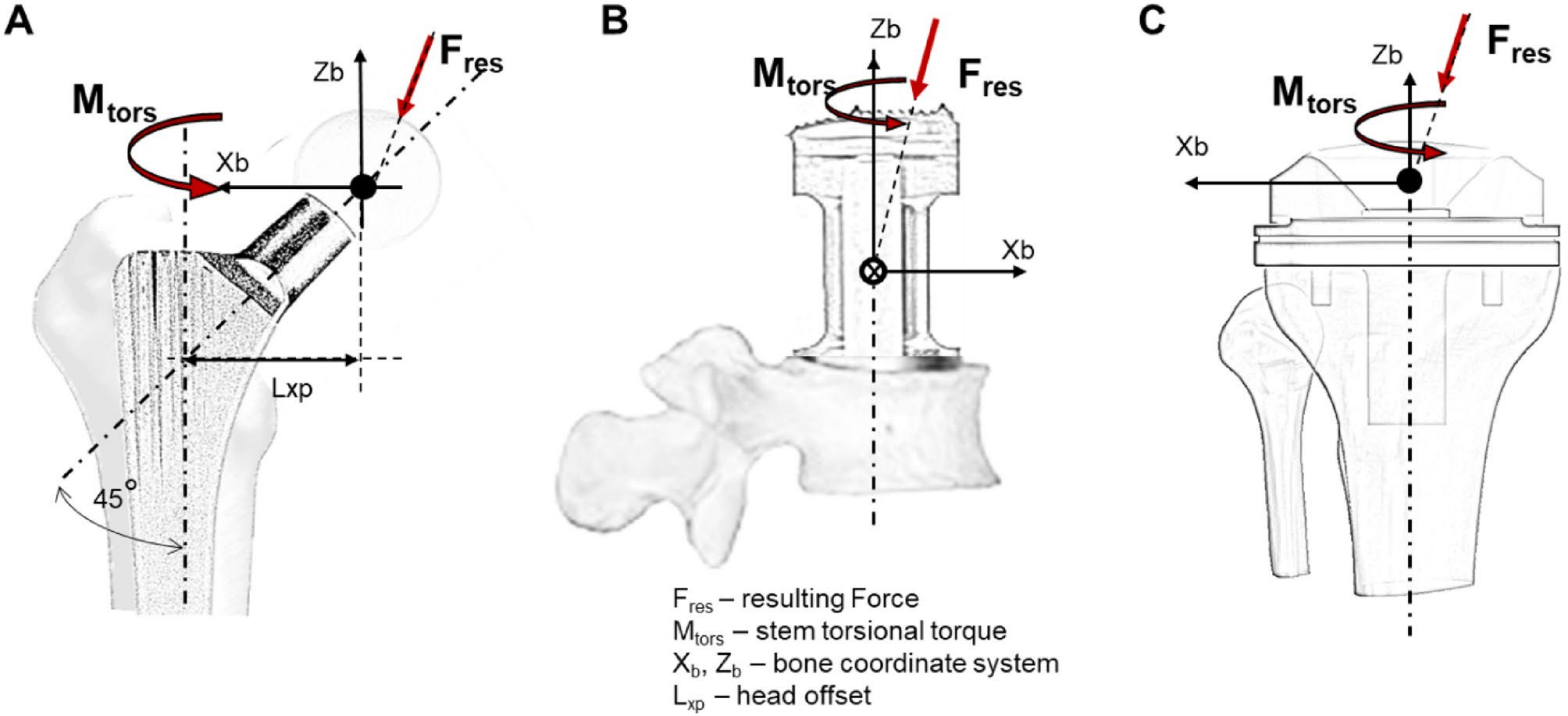
Coordinate system of the hip implant (**A**), the vertebral body replacement (**B**), and the knee implant (**C**) relative to bone and in vivo load measurements as the resultant contact force (F_res_) and torsional torque (M_tors_). The arrows indicate the direction of a positive force and torque.

The torsional torque M_tors_ in VBR and KI is equal to the moment M_z_ in the bone coordinate system for both implants since the implant axis of VBR and KI is the z-axis in the bone coordinate system. The torsional torque M_tors_ in HI acts around the stem axis of the implant, which is distant from the implant axis by head offset. The implant-specific head offset is calculated from the known length L and angle α = 45° of the implant neck (L_xp_, Fig. 1, part A) by the following equation.

$${M}_{tors}= {M}_{z}- {F}_{y}*{L}_{xp}$$

Figure 1 shows the coordinate system of HI, VBR, and KI. The resultant forces are reported as a percentage of the individual’s body weight (%BW) and corresponding the torsional torques are reported as %BW*m.

The duration of the standing phase was variable in different participants and repetition trails. Therefore, the start and end points of lifting up and putting down were determined from the videos and considered for the analysis. These so-called load-time patterns of the simultaneously recorded in vivo load measurements were first averaged individually for each participant^35^. Subsequently, the mean of the curves obtained of all investigated participants were calculated per implant type. Additionally, the peak forces were extracted. Note that the peak forces in the mean load patterns can slightly deviate from their averaged numbers due to the fact that peaks were timed differently between participant or trails.

The videos for posture classification were mainly recorded from a frontal perspective, but some also recorded from an oblique frontal or lateral perspective. The perspective of the videos was judged by an experienced ergonomist as suitable for the posture classification of the simple lifting task according to the observational methods used. The postures of the participants were viewed frame-by-frame on the videos and classified as defined by OWAS^9^ and REBA^10^. The postures to be classified for this study involve simple and repetitive postures of the lifting tasks, which entail less variance than in standard industrial work activities, so the classification was carried out by one experienced observer. A second experienced observer sample checked the observations and found no deviation from his classification. After the posture classification, the corresponding action levels of OWAS (OWAS-AL) and REBA (REBA-AL) were assigned frame-by-frame. The AL are presented in distributions. A data matrix was generated to merge F_res_, M_tors_, OWAS-AL, and REBA-AL, this means that for each frame, both the measured values for F_res_ and M_tors_ as well as the observed OAWS-AL and REBA-AL are recorded. For comparison of in vivo load measurements with OWAS-AL or REBA-AL mean values of F_res_ and M_tors_ were calculated over the respective OWAS-AL and REBA-AL.

### Statistical analysis

The question to be investigated is whether there are differences in F_res_ and M_tors_ between different OWAS-AL and REBA-AL. A student’s t-tests (used if two AL occurred) and a univariate ANOVA with Bonferroni corrected post-hoc tests (used if more than two AL occurred) were used to test for group differences of F_res_ and M_tors_ in OWAS-AL and REBA-AL. The comparison between stoop and squat lifting was performed descriptively. Statistical analyses were performed with IBM SPSS Statistics for Windows in version 28.0.1 (IBM Corp., 2021, Armonk, United States of Amercia). Type one error probabilities were accepted at an α-level of *p* = 0.05.

## Results

### In vivo load measurements

**Figure 2:**
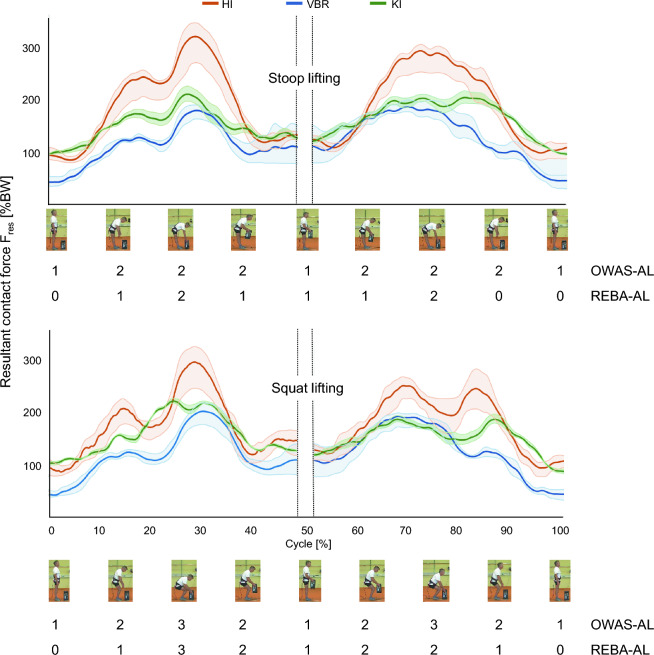
Curve of 25%-, 50%-, and 75%-percentile in vivo resultant contact forces F_res_ of the hip (HI), spine (VBR), and knee (KI) during stoop (upper part) and squat lifting (lower part) and OWAS-AL and REBA-AL for the example video frames shown along the entire lifting cycle.

**Figure 3:**
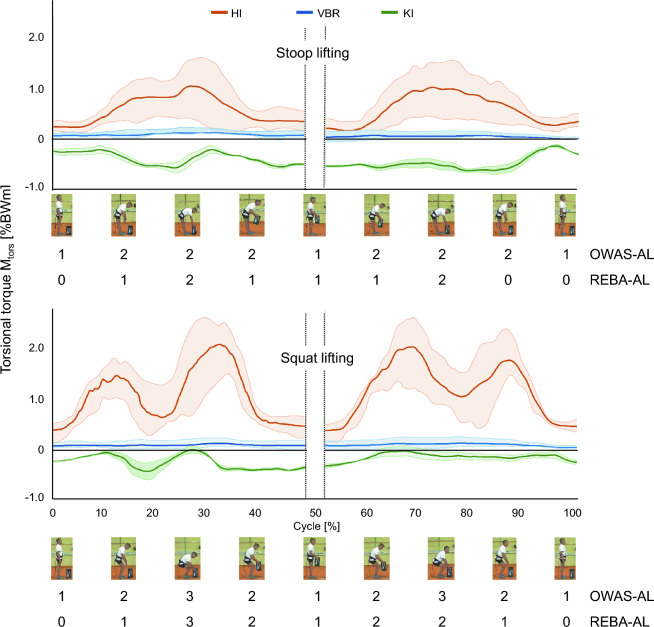
Curve of 25%-, 50%-, and 75%-percentile in vivo torsional torques M_tors_ of the hip (HI), spine (VBR), and knee (KI) during stoop (upper part) and squat lifting (lower part) and OWAS-AL and REBA-AL for the example video frames shown along the entire lifting cycle.

The in vivo load measurements are shown in mean curves of resultant contact force F_res_ (Fig. 2) and of the torsional torque M_tors_ (Fig. 3). The mean values of F_res_ for HI in the stance phase are at around 85–100%BW and reach a maximum of 321%BW during stoop lifting and 294%BW during squat lifting. The mean values F_res_ for KI in stance are approximately in the same range as those in stance phase for HI at around 90–100%BW. F_res_ (KI) reaches a maximum of 210%BW during stoop lifting and 218%BW during squat lifting. The mean values of F_res_ for VBR in stance phase are generally lower than the values in HI and KI with about 40%BW in stance phase, 186%BW at maximum during stoop lifting, and 201%BW at maximum during squat lifting.

The mean values of M_tors_ are highest in HI compared to VBR and KI. At HI they range between 0.2–0.4%BWm in stance phase, 1.0%BWm at maximum during stoop lifting, and 2.1%BWm at maximum during squat lifting. VBR showed the smallest torsional moments compared to HI and KI. During the stance phase the mean value of M_tors_ at VBR is 0.01%BW, whereas a maximum of 0.10%BWm is reached during stoop lifting and 0.11%BWm during squat lifting. The mean value of M_tors_ at KI range between 0.1%BW in stance phase and 0.6%BWm during stoop lifting or 0.5%BWm during squat lifting.

### Distributions of OWAS and REBA action levels

The results are based on 80,752 frame-by-frame observations according to OWAS and REBA, respectively. The overall distribution of the OWAS-AL reveal that 41.1% of the observations are assessed with AL1, 35.4% are assessed with AL2, and 23.5% are assessed with AL3. The distributions of OWAS-AL related to lifting technique, implant, and their combinations are shown in Table 2.

**Table 2:** Distributions of OWAS-AL related to lifting technique, implant, and their combinations.

	Lifting technique	
	Stoop lifting (43,065 observations) AL1 = 42% AL2 = 58%	Squat lifting (37,687 observations) AL1 = 41% AL2 = 9% AL3 = 50%
Implant		
HI (43,129 observations) AL1 = 40% AL2 = 33% AL3 = 27%	21,475 observations AL1 = 42% AL2 = 58%	21,654 observations AL1 = 39% AL2 = 8% AL3 = 53%
VBR (19,775 observations) AL1 = 43% AL2 = 38% AL3 = 18%	11,990 observations AL1 = 43% AL2 = 57%	7,785 observations AL1 = 44% AL2 = 9% AL3 = 47%
KI (17,848 observations) AL1 = 41% AL2 = 38% AL3 = 22%	9,600 observations AL1 = 40% AL2 = 60%	8,248 observations AL1 = 41% AL2 = 12% AL3 = 47%

HI = hip implant, VBR = vertebral body replacement, KI = knee implant, AL1 = action level 1, AL2 = action level 2, AL3 = action level 3.

The overall distribution of the REBA-AL reveal that 21.1% of the observations are assessed with AL0, 38.3% are assessed with AL1, 39.2% are assessed with AL2, and 1.4% are assessed with AL3. The distributions of REBA-AL related to lifting technique, implant, and their combinations are shown in Table 3.

**Table 3:** Distributions of REBA-AL related to lifting technique, implant, and their combinations.

	Lifting technique	
	Stoop lifting (43,065 observations) AL0 = 20% AL1 = 39% AL2 = 41%	Squat lifting (37,687 observations) AL0 = 22% AL1 = 38% AL2 = 37% AL3 = 3%
Implant		
HI (43,129 observations) AL0 = 21 AL1 = 39 AL2 = 38 AL3 = 2	21,475 observations AL0 = 21% AL1 = 39% AL2 = 40%	21,654 observations AL0 = 21% AL1 = 39% AL2 = 35% AL3 = 5%
VBR (19,775 observations) AL0 = 21 AL1 = 38 AL2 = 40 AL3 = 1	11,990 observations AL0 = 19% AL1 = 40% AL2 = 41%	7,785 observations AL0 = 25% AL1 = 36% AL2 = 38% AL3 = 1%
KI (17,848 observations) AL0 = 21 AL1 = 36 AL2 = 43	9,600 observations AL0 = 19% AL1 = 36% AL2 = 45%	8,248 observations AL0 = 24% AL1 = 36% AL2 = 40%

HI = hip implant, VBR = vertebral body replacement, KI = knee implant, AL0 = action level 0, AL1 = action level 1, AL2 = action level 2, AL3 = action level 3.

The distributions of the lowest action level, OWAS-AL1 and REBA-AL0, vary only slightly depending on lifting technique, implant, and their combinations. The distributions of the higher AL show that OWAS assesses the risk for squat lifting higher than for stoop lifting, whereas REBA assesses the risk equally or only slightly higher for squat lifting compared to stoop lifting.

### Comparison of in vivo load measurements with OWAS-AL or REBA-AL

The statistical analysis reveals significant differences in F_res_ and M_tors_ depending on OWAS-AL with medium to high effect sizes, except for M_tors_ in KI during stoop lifting and the post-hoc test of F_res_ between OWAS-AL1 and OWAS-AL2 in HI during squat lifting. For the significant effects, mean values of F_res_ and M_tors_ increase with increasing OWAS-AL, except for M_tors_ in KI during squat lifting. The results of the statistical analysis for OWAS-AL are shown in Table 4.

**Table 4:** Differences in mean values (standard deviation (SD)) of resultant contact force (F_res_) and torsional torque (M_tors_) between OWAS-AL with T-test results and Cohen’s d effect size or ANOVA results and η^2^ effect size.

	Stoop lifting		Squat lifting		
	OWAS-AL1	OWAS-AL2	OWAS-AL1	OWAS-AL2	OWAS-AL3
F_res_ (SD) [%BW]					
HI	117(27)	211(80)	123(37)	124(37)	195(68)
	*p* < 0.01, d = 1.48		*p* < 0.01*, η^2^ = 0.29		
KI	118(19)	165(38)	112(20)	128(21)	172(33)
	*p* < 0.01, d = 1.50		*p* < 0.01, η2 = 0.53		
VBR	63(37)	123(43)	57(41)	84(34)	138(50)
	*p* < 0.01, d = 1.49		*p* < 0.01, η2 = 0.43		
M_tors_ (SD) [%BWm]					
HI	0.28(0.21)	0.63(0.55)	0.38(0.26)	0.55(0.32)	1.38(0.85)
	*p* < 0.01, d = 0.81		*p* < 0.01, η2 = 0.36		
KI	0.44(0.13)	0.44(0.23)	0.33(0.12)	0.25(0.13)	0.16(0.15)
	*p* = 0.78, d = 0.01		*p* < 0.01, η2 = 0.28		
VBR	0.10(0.05)	0.15(0.05)	0.10(0.04)	0.12(0.04)	0.15(0.05)
	*p* < 0.01, d = 0.99		*p* < 0.01, η2 = 0.21		

(HI = hip implant, VBR = vertebral body replacement, KI = knee implant, OWAS-AL1 = OWAS action level 1, OWAS-AL2 = OWAS action level 2, OWAS-AL3 = OWAS action level 3, * = post-hoc test between OWAS-AL1 and OWAS-AL2 not significant).

The statistical analysis reveals significant differences in F_res_ and M_tors_ depending on REBA-AL with medium to high effect sizes, except for the post-hoc test of M_tors_ between REBA-AL0 and REBA-AL1 in HI during squat lifting. For the significant effects, mean values of F_res_ and M_tors_ increase with increasing REBA-AL, except for F_res_ and M_tors_ in KI during squat lifting. The results of the statistical analysis for OWAS-AL are shown in Table 5.

**Table 5:** Differences in mean values (standard deviation) of resultant contact force (F_res_) and torsional torque (M_tors_) between REBA-AL with T-test results and Cohen’s d effect size or ANOVA results and η^2^ effect size.

	Stoop lifting			Squat lifting			
	REBA-AL0	REBA-AL1	REBA-AL2	REBA-AL0	REBA-AL1	REBA-AL2	REBA-AL3
F_res_ [%BW]							
HI	107(29)	145(51)	231(77)	106(34)	139(39)	209(67)	231(54)
	*p* < 0.01, η2 = 0.42			*p* < 0.01, η2 = 0.42			
KI	104(10)	131(22)	177(34)	100(18)	129(21)	178(30)	166(3)
	*p* < 0.01, η2 = 0.55			*p* < 0.01, η2 = 0.62			
VBR	38(17)	84(35)	140(36)	46(36)	83(45)	143(49)	173(28)
	*p* < 0.01, η2 = 0.58			*p* < 0.01, η2 = 0.45			
M_tors_ [%BWm]							
HI	0.31(0.22)	0.37(0.35)	0.68(0.60)	0.41(0.28)	0.62(0.50)	1.47(0.91)	1.66(0.78)
	*p* < 0.01, η2 = 0.12			*p* < 0.01, η2 = 0.34			
KI	0.35(0.11)	0.41(0.18)	0.51(0.21)	0.28(0.10)	0.29(0.16)	0.18(0.26)	0.36(0.00)
	*p* < 0.01, η2 = 0.11			*p* < 0.01*, η^2^ = 0.12			
VBR	0.07(0.03)	0.13(0.04)	0.16(0.05)	0.09(0.04)	0.12(0.04)	0.15(0.05)	0.18(0.07)
	*p* < 0.01, η2 = 0.40			*p* < 0.01, η2 = 0.22			

(HI = hip implant, VBR = vertebral body replacement, KI = knee implant, REBA-AL0 = REBA action level 0, REBA-AL1 = REBA action level 1, REBA-AL2 = REBA action level 2, REBA-AL3 = REBAaction level 3, * = post-hoc test between REBA-AL0 and REBA-AL1 not significant).

## Discussion

This study aimed to increase the knowledge on the validity of ergonomic observational methods by comparing OWAS and REBA with in vivo load measurements at hip, spine, and knee. A MMH activity applying stoop and squat lifting technique were selected as work activities to be investigated. The result reveals that in most conditions OWAS-AL and REBA-AL significantly differ from mean values of the in vivo load measurements at hip, spine, and knee. More precisely, an increasing OWAS-AL or REBA-AL leads to an increase in F_res_ and M_tors_. Thus, OWAS and REBA can distinguish between different biomechanical exposures at hip, spine, and knee. It can be concluded that both observational methods can distinguish between different in vivo loads acting at hip, spine, and knee during the investigated MMH. Although we are not aware of a comparative study, studies that simultaneously compared outcomes of biomechanical models with OWAS or REBA were able to draw similar conclusions^15,20^.

The sensitivity for data collection and ergonomic assessment of the two observation methods is conceptually different. OWAS uses broader posture categories and action levels compared to REBA, whereas REBA allows for a more sophisticated analysis compared to OWAS. However, both methods were able to indicate differences of in vivo loads during the MMH activity. However, the results show that between the two lifting techniques, equal AL sometimes do not provide the same mean values of the in vivo load. For example, F_res_ for OWAS-AL2 at HI during stoop lifting (211%BW) is greater than for OWAS-AL3 during squat lifting (195%BW) and F_res_ at KI for REBA-AL2 during stoop lifting (177%BW) is greater than for REBA-AL3 during squat lifting (166%BW). One explanation may be that, for example, it cannot be clearly determined until when an actual load of less than 10 kg is effective, and from which point onwards it is effective above 10 kg. Observers will basically either underestimate or overestimate the risk as defined by the observational method by rating the load when the box is no longer touching the ground or when the hands are gripping the box. The frame-by-frame data show that in this study the observer overestimates the risk. In such a case, however, the typical application of OWAS and REBA in operational practice by means of time sampling leads to the fact that the working postures to be observed are classified individually and therefore, tend to be also overestimate, because they are perceived independently of each other. Another example can be found in basic methodical issues. As Li and Buckle^6^ mentioned earlier, that little is known about the relative importance of each risk factor. In addition, it requires knowledge of which intensity and which temporal characteristics of exposures of risk factors increase a local and the overall risk. According to^36^, both peak and cumulative load are relevant risk factors for evaluating work activities during MMH. Furthermore, they represent independent risk factors for the occurrence of WMSD^37^. However, cumulative load is only partially represented by the observational methods. Current research continues to reveal a relationship between cumulative workload and the occurrence of WMSD^38^ and is also increasingly (re)addressing the modelling of the exposure to this risk factor^39,40^.

Comparing the lifting technique, OWAS results reveal that the risk for WMSD of squat lifting is clearly higher than the risk for stoop lifting, whereas REBA results reveal that the risk of squat lifting is slightly higher or the same than the risk for stoop lifting. Peak compression force on the lumbar spine is the main biomechanical criterion for evaluating lifting of low-lying objects^36^. From this point of view, F_res_ for VBR reveals higher values for squat lifting compared to stoop lifting. The results of this study overall suggest that squat lifting has a higher risk than stoop lifting, which seems uncommon at first because squat lifting seems to be widely accepted as the "correct" way of lifting^41^. However, there is no consistent evidence of the effectiveness of a particular lifting technique, and it is known that no single lifting technique can be advised for all lifting conditions^42–46^. Studies may show that the actual lifting technique may be less important than the lifting conditions, such as load placement, time pressure and experience, or small measures like shifting or tilting the load^45,47–50^. Since the NIOSH lifting equation is not intended to evaluate differences between lifting techniques^25^, based on the results of this study, it might be an appropriate approach to evaluate with OWAS or REBA instead of the NIOSH lifting equation when manual material handling is performed with a high variance in lifting technique. Latest research reveal that real-time feedback of such underlying parameters, especially outcomes of biomechanical models, can improve the lifting technique in MMH^51,52^.

The study results must always be interpreted against the limitation that this is a secondary data analysis, because a part of the data was previously published under other aims^32^. The classification of OWAS and REBA is based on videos, most of which were recorded from a frontal perspective and some from a frontal-lateral or lateral perspective. Furthermore, the special sample of participants must be mentioned. The participants all had indications for musculoskeletal disorders, which were treated by artificial implants. Even though we did not notice any remarkable differences in the movements performed (e.g., unusual movement strategies, range of motion restrictions, etc.) compared to healthy people, we cannot exclude the possibility that a corresponding systematic error is present in the data. When interpreting the results, the small sample size, especially for KI, must be taking into account. The values at HI and KI can be interpreted absolutely because they take the same acting load compared to a natural joint. The values at VBR cannot be interpreted absolutely because VBR do not take the same acting load compared to a natural vertebra^53^. However, ratios of changes between different postures in the acting load can be considered equal. In addition, it should be noted that a joint implant is a replica of a natural joint. Accordingly, generalizations of the study results are to be made regarding these aspects.

Finally, it should be noted that when selecting an appropriate method, validity is an important, however, not the only important criterion^8^. In addition, the definition of a sampling strategy providing reliable values obtained through observation is as least as important as a reasonable decision of selecting an appropriate method^13^.

## Conclusion

The study results reveal that action levels of the observational methods, OWAS and REBA, can distinguish between different in vivo loads at hip, spine, and knee during the investigated MMH activities. However, the results show that between the two lifting techniques, stoop and squat lifting, the same action levels of OWAS and REBA do not necessarily provide equal mean values of in vivo load. This seems to be a relevant side finding that addresses fundamental issues in ergonomics science. Further research on criterion validity could, for example, systematically vary the validation reference as a statistically independent variable and examine the variance that arises in the action levels of observational methods. Furthermore, the study results based on in vivo load measurements of spine, hip and knee, and observational methods OWAS and REBA for assessing exposure to risk factors for work-related musculoskeletal disorders suggest that the performed squat lifting techniques had a higher risk for WMSD than the performed stoop lifting techniques. The question of the “correct” lifting technique and which lifting technique should be taught (when) as proper has challenged the scientific community so far and will continue to do so.

## Data Availability

The datasets generated and analysed during the current study are public available in OrthoLoad repository, on the non-commercial website www.orthoload.com.
